# Can sleep affect destination memory? A prospective narrative review

**DOI:** 10.3389/fpsyg.2025.1558110

**Published:** 2025-03-14

**Authors:** Tanisha Rathore, Gunjan Joshi, Kedarmal Verma

**Affiliations:** ^1^Sleep-Cognition and Psychology Experimental Laboratory, Indore, India; ^2^School of Humanities and Social Sciences, Indian Institute of Technology Indore, Indore, India

**Keywords:** destination memory, source memory, social communication, efficacy, pre-frontal cortex, medial temporal lobe

## Abstract

The destination memory is the ability of individuals to remember to whom the information has been delivered. The memory system is an essential and critical piece of social communication and human social cognition. Previously, research has been done on the relationship between source memory and sleep which are critical and fundamental processes influencing our daily lives. However, this present prospective narrative review sheds light on the idea of beginning new research to understand the relationship between destination memory and sleep. Although no such literature exists that can explain this relationship, this review will try to understand prospective future directions by synthesizing available literature on sleep and the source memory. The present narrative review provides an overview of research executed in the fields of source memory, destination memory, and sleep. Destination memory and source memory are the opposite sides of the same coin. They are a part of the episodic memory system. Research suggests that they share similarities, namely their contextual nature, involvement of the pre-frontal cortex (PFC), and role of the medial temporal lobe (MTL). Studies on the effect of sleep on source memory have indicated that it plays a role in consolidation. This consolidation involves binding the item to its context. Due to the shared characteristics of source and destination memory, it can be suggested that sleep may play a role in influencing destination memory. Understanding this relationship will have implications for enhancing social memory/communication efficacy through sleep hygiene.

## Introduction

Memory has a significant impact on our social lives. It is challenging to conceive of any discourse that does not rely on the utilization of prior recollections in any capacity. This ability helps strengthen interpersonal connections. It has already been seen that memory impairments in older people with memory-related disorders including dementia and Alzheimer’s have detrimental effects on their social life and in turn lead to reduced wellbeing (Shell, 2015). In our lives, we might have found ourselves in situations where we remember telling someone a story only to find out later that we did not, or we relayed the information to a different receiver. This is how individuals make communication errors in daily life which might result in the feeling of embarrassment and this we can call a distorted destination memory. Destination memory is the ability to remember with whom we share specific information. Such instances of miscommunication can lead to serious mishaps in both personal and professional life. For instance, what if you get mad at your partner for not bringing groceries for an important dinner? But you shared that you need groceries for dinner with your friend. These mistakes could end up hampering your relationships. Research has shown that destination recall is difficult and could be subject to many distortions. Destination memory can be affected by many factors like aging (Gopie et al., 2010; Haj et al., 2015), diseases like Alzheimer’s (El Haj et al., 2015c), self (Gopie et al., 2010; Gopie and MacLeod, 2009), familiarity (Haj et al., 2015), attractiveness of the receptor (El Haj and Ndobo, 2021). This interesting memory system plays an integral part in social communication and its efficacy. On the other side, sleep is very important for better cognition, and good health. Research on memory and sleep shows that sleep can both enhance and diminish our memory (Takashima et al., 2006; Van Der Helm et al., 2011). The process of binding in memory during sleep helps in consolidation and a lack of it will negatively influence consolidation.

In our review, we identify commonalities in how episodic memory, source memory, and destination memory operate, hinting at potential sleep-related effects on destination memory. We observe shared features such as their reliance on contextual information (Gopie and MacLeod, 2009), involvement of the pre-frontal cortex (PFC) (Mitchell et al., 2004; Slotnick et al., 2003; Haj et al., 2014), and the role of the medial temporal lobe. Additionally, we explore the reciprocal influence between sleep and communication, highlighting how sleep deprivation can hinder social interactions, while effective communication can impact sleep quality, particularly in the context of supportive relationships (Beattie et al., 2015). Lastly, we underscore the vital role of sleep in consolidating both episodic and source memory, which could extend to its influence on destination memory (Van Der Helm et al., 2011; Olsen et al., 2012).

## Search strategy

The search query incorporated the following keywords: “destination memory,” “sleep,” “communication,” and “memory consolidation.” The Boolean operator “AND” was employed to use these search terms in SCOPUS and PubMed to find scholarly papers that include all of them. Filters were applied to narrow down the search results based on the publication date, specifically within the range from 2000 to 2023. This review also included significant papers identified through backward referencing. After retaining relevant material, excluding duplicates from both databases, and adding a few sources that were found manually a total of 48 texts were included in the brief review (Table 1).

**Table 1:** Search outcomes of articles.

Serial no.	Keywords	SCOPUS	Pub Med
1	Destination memory	70	301
2	Destination memory AND sleep	0	0
3	Sleep AND communication	598	700
4	Sleep AND memory consolidation	3,313	2,404

Total sources included: 48.

## History of destination memory

The ground-breaking study of Koriat, Ben-Zur, and Sheffer, who examined the prevalence of action repeats in old age, established the idea of destination memory (e.g., repeating the same action, telling the same story over and over) (Koriat et al., 1988). Young and elderly individuals were asked to remember as many words as they could from a list of terms that had previously been investigated by these researchers. The older adults recalled fewer words from the list than the younger individuals, but showed a stronger propensity to repeat the phrases they had retained. In another study, Koriat found that older adults had poorer integration of information with its encoding context, which they believed to be the cause of “output monitoring” impairments in normal aging. The idea of “output monitoring” is comparable to “target memory” (Koriat et al., 1991). The latter idea was put out by Marsh and Hicks who contrasted the recall of the source of an object (also known as “source memory”) with the recipient of the object (also called “target memory”) (Marsh and Hicks, 2002). In a similar spirit, the term “target monitoring” was coined by Brown, Hornstein, and Memon to describe the capacity to control who is informed of information (Brown et al., 2006). In the study, target monitoring was evaluated by inviting young volunteers to provide personal information in exchange for pictures of celebrities for four sessions spaced over one day. It was forbidden for participants to repeat information to the same celebrity across many sessions. Unsurprisingly, the findings revealed that participants found it harder to prevent duplications in the last session than in the others. Gopie and MacLeod in 2009 came up with a new fascinating term “destination memory” to describe the ability to remember to whom we have shared previous information (Gopie and MacLeod, 2009).

## How are source, destination, and episodic memory related

The episodic memory system, first defined by Tulving, includes both source memory and destination memory (Tulving, 2002). The Oxford Dictionary defines it as a kind of long-term memory that involves the conscious recall of past events along with their context, such as time, place, emotions they were experienced with, etc. Source memory is defined as a memory of the episodic source from which a certain idea or knowledge was learned (Schacter et al., 1991). The work done on source memory has explored its relationship with aging (Schacter et al., 1991), development (Drummey and Newcombe, 2002) and emotions (Doerksen and Shimamura, 2001). It is the capacity to recall the source from which information was obtained. In a similar vein destination memory is the ability to remember to whom we shared specific information. Current research on destination memory has investigated its relationship with various variables including familiarity, attractiveness, theory of mind, age, gender, etc. (El Haj et al., 2020; El Haj et al., 2015a; El Haj et al., 2015b; El Haj and Ndobo, 2021; Kladi et al., 2022).

It is possible to make out the relationships between how source memories and destination memories function. First, think about how destination memory and source memory are contextual. Both have an episodic quality, as they rely on context (like time and place) (Gopie and MacLeod, 2009). According to the Destination Memory Framework (DMF) proposed by Haj, contextual processing is crucial for destination memory, as recall depends on reconstructing the encoding context (El Haj and Miller, 2018), and the contextual processing is a again the important part of source memory (Doerksen and Shimamura, 2001; Raj and Bell, 2010). Secondly, the involvement of the pre-frontal cortex (PFC) is another similarity. Few fMRI studies have shown that source memory tasks activate regions in the PFC (Mitchell et al., 2004; Slotnick et al., 2003). The cognitive resources underlying destination memory involve episodic recall, executive functioning, and self-referential processes (Haj et al., 2014). The role of executive functioning is to either allow the formation of association between the appropriate encoding context known as *binding* or to restrict from an inappropriate association to be formed known as *inhibition* (El Haj and Miller, 2018), and this binding of information helps in the consolidation of memory (Yonelinas et al., 2019). It was investigated that the contextual processing uses the executive functions of our brain suggesting a role of the pre-frontal cortex (El Haj and Miller, 2018). In neuroimaging research, the PFC has been linked to the binding of items and contexts (Mitchell and Johnson, 2009; Ranganath, 2010). Lastly, both memory types involve the medial temporal lobe activity. Gold found that patients with hippocampal damage performed worse than healthy patients on a source memory task (Gold et al., 2006), whereas the fMRI study showed the higher destination memory recognition task scores were linked to more activity in the para hippocampal gyrus (Mugikura et al., 2016).

## Sleep and communication

Sleep and communication are both essential aspects of human life, and their relationship is complex. We can understand their relationship by explaining when we are sleep-deprived, our communication skills can be affected (Beattie et al., 2015). Sleep deprivation can impact social interaction since communicators may feel irritable, moody, or have difficulty concentrating, which can negatively impact our social interactions (Beattie et al., 2015). We may also be less likely to engage in social activities if we are too tired. Lack of sleep can make it harder for us to concentrate, process information, and remember details, which can impact our ability to communicate effectively.

Contrary to the effect of sleep on communication, we can understand how communication between people can affect sleep hygiene and quality. Sleep hygiene refers to habits and practices that promote good sleep. Effective communication and good social relationships can help us establish healthy sleep habits and promote better sleep hygiene (Kent et al., 2015). Another study found that aversive relationships with stressful communication predicted poorer sleep quality, whereas supporting relationships were favorably correlated with it; these effects were most prominent in close relationships (Kent et al., 2015).

Understanding the relationship between destination memory and sleep is crucial, as both significantly impact social interactions (El Haj, 2022). This investigation may deepen our grasp of the complex relationship between sleep and productive communication by improving our understanding of how sleep patterns impact our capacity to remember and retain the specific information we have communicated with others.

## Sleep and memory consolidation

The relationship between sleep and memory has been well documented. Research indicates that sleep aids in the consolidation of both declarative and non-declarative memory (Diekelmann and Born, 2010; Asp et al., 2022; Rångtell et al., 2017). Sleep has been shown to affect the performance of episodic memory (Van Der Helm et al., 2011), whether it is the reduced decay (Takashima et al., 2006), or the enhancement of episodic memory because of sleep. According to the Standard Systems Consolidation Theory (SSCT), the first phase of memory consolidation starts after the initial minutes of learning, with the hippocampus playing an important role. In the next phase, memory is further consolidated during offline periods, including sleep, even in the absence of direct hippocampal involvement (Dudai, 2004; Yonelinas et al., 2019). This theory suggests that memory consolidation is a process that is done both when we are awake and even through sleep.

Binding is an important function of the brain that links different neural representations together that can be used further for memory, decision making and perception (Zimmer et al., 2006). Several studies have showed that binding in individuals is negatively affected by sleep deprivation (Harrison and Horne, 1999; Honn et al., 2019; Killgore et al., 2006; Mckenna et al., 2007; Whitney et al., 2015). Existing literature states that Total Sleep Deprivation (TDS) is detrimental to source memory binding. The hippocampus has been found to show a decline in activity as a result of TDS resulting in impairment of the episodic memory (Chai et al., 2020; Van Der Helm et al., 2011; Van Der Werf et al., 2009; Yoo et al., 2007). Similar to how sleep facilitates memory binding, the hippocampus also supports this process during wakefulness in source memory encoding (Olsen et al., 2012). Neuroimaging studies performed on patients and healthy individuals have shown that the PFC is involved in the binding of items and contexts (Mitchell and Johnson, 2009; Ranganath, 2010). As a result, patients with frontal lobe lesions who perform similarly to control participants in terms of item memory still display deficits in source memory when compared to controls (Janowsky et al., 1989). The frontal and hippocampus areas interact to help build and retrieve episodic memories (Woodcock et al., 2015) and it has been observed that source memory tends to use the functional connections between the hippocampus and PFC more often than item memory (Monge et al., 2018) implying that source memory may be affected adversely by TSD (Ranganath, 2010; Rubin et al., 2017).

## Future directions

For researchers venturing into this field, the potential focus lies in the exploration of destination memory and its intricate connections with subjective sleep. Subjective sleep entails an individual’s personal perception of their sleep quality, gauged through self-report questionnaires. While, objective sleep involves the quantifiable physiological aspects of sleep, assessed via methods like polysomnography, sleep monitors, and actigraphy, among others. Investigating these dimensions can shed light on the pivotal role sleep plays in the realm of destination memory and, more broadly, its impact on communication.

These are some possible avenues investigators may take in the future to learn more about this connection:

### Longitudinal study

These studies will help track changes in destination memory over time. Additionally, these studies will also help determine how destination memory is related to retention intervals. These studies may assist in discovering patterns and modifications in memory retention that relate to the quality and length of sleep if we are able to take sleep into consideration.

### Sleep stage-specific effects

Sleep is an essential component in the process of integrating new information into previously stored memories. Additionally, the different stages of sleep each have a unique role in the consolidation process. It will be highly intriguing to study how various specialized stages of sleep, such as REM (rapid eye movement), SWS (slow-wave sleep), spindles, and other sleep parameters, affect the consolidation of destination memory.

### Relative sleep timing

An interesting possibility for future study is to investigate how the timing of one’s sleep affects the encoding of one’s destination memory. Researchers will be able to evaluate whether sleep timing (immediate or after delayed encoding) impacts destination memory performance.

### Neural mechanism

In the future, it could be a good idea to research the brain mechanisms that are responsible for the relationship between sleep and destination memory. Researchers in the future will use neuroimaging techniques, such as functional magnetic resonance imaging (fMRI) or electroencephalography (EEG), to find the parts of the brain and network activity that are linked to making destination memories while a person is asleep.

### Sleep disorders

Investigators may consider the potential impact of various sleep-related conditions, such as insomnia, sleep apnea, and others, on destination memory.

By exploring these possible paths, researchers may learn more about the complex relationship between sleep and destination memory. This could lead to useful insights into how to best use sleep to improve memory retention in a wide range of situations.

## Limitations and proposed studies

While the current review synthesizes existing literature on sleep, source memory, and destination memory to propose a theoretical relationship, we acknowledge the lack of direct empirical evidence. To address this limitation and strengthen the empirical foundation of this research area, we propose the following pilot studies:

### Sleep-dependent consolidation of destination memory

A controlled sleep deprivation study could significantly enhance our understanding of destination memory by comparing performance between well-rested participants and those deprived of sleep. This approach would allow researchers to assess the necessity of sleep for accurately recalling the recipients of information, which is crucial for effective social interactions. Previous research has established that sleep plays a vital role in memory consolidation, particularly during slow-wave sleep (SWS) and rapid eye movement (REM) stages, where memories are reactivated and integrated into long-term storage (Fan et al., 2024). Sleep deprivation has been shown to impair binding processes in memory, leading to difficulties in associating information with its context, which is essential for destination memory (Kurinec et al., 2021). Moreover, studies indicate that sleep-deprived individuals exhibit declines in cognitive control and executive functions, which are critical for processing and recalling social interactions (Kim et al., 2022). By investigating how sleep deprivation affects destination memory specifically, researchers can elucidate the underlying mechanisms and potentially identify strategies to mitigate the negative impacts of sleep loss on social cognition and communication efficacy.

### Sleep stages and destination memory

Given the distinct roles of different sleep stages in memory consolidation, polysomnography-based studies could indeed provide valuable insights into how specific sleep stages affect destination memory retention. Research has shown that slow-wave sleep (SWS) is particularly important for declarative memory consolidation, while REM sleep plays a role in procedural and emotional memory processing (Rasch and Born, 2013). A polysomnography study could examine whether SWS duration or quality correlates with improved destination memory performance, given its contextual nature similar to other declarative memories. Additionally, the study could investigate if REM sleep contributes to the emotional or social aspects of destination memory. By analyzing sleep spindles, which are associated with memory consolidation during NREM sleep, researchers could explore their relationship with destination memory retention (Marshall et al., 2020). Such a study would not only shed light on the sleep-dependent mechanisms of destination memory consolidation but also potentially inform strategies for enhancing this crucial aspect of social cognition through sleep optimization.

These proposed studies would provide direct empirical evidence on the relationship between sleep and destination memory, addressing the current limitation of relying on indirect evidence from source memory studies. By conducting these pilot studies, we can begin to establish a more robust empirical foundation for understanding how sleep affects destination memory, potentially opening new avenues for research in this area.

## Conclusion

The review here aims to put a spotlight on the relationship between sleep and source memory binding, where sleep has been shown to enhance the binding process (Takashima et al., 2006; Van Der Helm et al., 2011), and lack of sleep has found to deteriorate the process of binding (Chai et al., 2020; Van Der Helm et al., 2011; Van Der Werf et al., 2009; Yoo et al., 2007). As mentioned above, source memory and destination memory share certain similarities in their functionality. Whether that be the contextual element of both memories or the involvement of the pre-frontal cortex. Since both involve the binding of item and context, and keeping in mind that binding in source memory is facilitated through sleep we can assume that sleep can play an important role in destination memory encoding or retrieval. We suggest that researches in the future should explore the relationship between sleep and destination memory.

When we searched through a large number of papers in a variety of article indexing libraries, we were unable to locate any literature that offered an explanation of the relationship between sleep and destination memory. We were encouraged to conduct a prospective narrative review since there was a lack of availability. This review will provide us with an idea of how to begin research investigations with the goal of getting a better understanding of the link between sleep and destination memory.
